# Case management for patients with chronic systolic heart failure in primary care: The HICMan exploratory randomised controlled trial

**DOI:** 10.1186/1745-6215-11-56

**Published:** 2010-05-17

**Authors:** Frank Peters-Klimm, Stephen Campbell, Katja Hermann, Cornelia U Kunz, Thomas Müller-Tasch, Joachim Szecsenyi

**Affiliations:** 1 Department of General Practice and Health Services Research, University Hospital Heidelberg, Heidelberg, Germany; 2 National Primary Care Research and Development Centre, University of Manchester, Manchester, UK; 3 Institute of Medical Biometry and Informatics, University Hospital Heidelberg, Heidelberg, Germany; 4 Department of Psychosomatic and General Internal Medicine, University Hospital Heidelberg, Heidelberg, Germany

## Abstract

**Background:**

Chronic (systolic) heart failure (CHF) represents a clinical syndrome with high individual and societal burden of disease. Multifaceted interventions like case management are seen as promising ways of improving patient outcomes, but lack a robust evidence base, especially for primary care. The aim of the study was to explore the effectiveness of a new model of CHF case management conducted by doctors' assistants (DAs, equivalent to a nursing role) and supported by general practitioners (GPs).

**Methods:**

This patient-randomised controlled trial (phase II) included 31 DAs and employing GPs from 29 small office-based practices in Germany. Patients with CHF received either case management (n = 99) consisting of telephone monitoring and home visits or usual care (n = 100) for 12 months. We obtained clinical data, health care utilisation data, and patient-reported data on generic and disease-specific quality of life (QoL, SF-36 and KCCQ), CHF self-care (EHFScBS) and on quality of care (PACIC-5A). To compare between groups at follow-up, we performed analyses of covariance and logistic regression models.

**Results:**

Baseline measurement showed high guideline adherence to evidence-based pharmacotherapy and good patient self-care: Patients received angiotensin converting enzyme inhibitors (or angiotensin-2 receptor antagonists) in 93.8% and 95%, and betablockers in 72.2% and 84%, and received both in combination in 68% and 80% of cases respectively. EHFScBS scores (SD) were 25.4 (8.4) and 25.0 (7.1). KCCQ overall summary scores (SD) were 65.4 (22.6) and 64.7 (22.7). We found low hospital admission and mortality rates. EHFScBS scores (-3.6 [-5.7;-1.6]) and PACIC and 5A scores (both 0.5, [0.3;0.7/0.8]) improved in favour of CM but QoL scores showed no significant group differences (Physical/Mental SF-36 summary scores/KCCQ-os [95%CI]: -0.3 [-3.0;2.5]/-0.1 [-3.4;3.1]/1.7 [-3.0;6.4]).

**Conclusions:**

In this sample, with little room for improvement regarding evidence-based pharmacotherapy and CHF self-care, case management showed no improved health outcomes or health care utilisation. However, case management significantly improved performance and key intermediate outcomes. Our study provides evidence for the feasibility of the case management model.

**Trial registration number:**

ISRCTN30822978

## Background

Chronic (systolic) heart failure (CHF) is a "common, disabling, deadly, and costly disease" [1] characterised by hospital admissions due to clinical deterioration. New treatment and care strategies focus on the prevention of admissions and improvement of prognosis. Effective knowledge transfer, for example the adherence of physicians to clinical practice guidelines (CPG) [2] and patients to treatment regimens [3], is regarded as a key issue for better patient outcomes.

For improved outcomes, multifaceted interventions are regarded as essential and many approaches have been tested. To foster comparability between the related studies, a taxonomy for chronic disease/case management has been suggested accounting for 8 domains (i.e. Patient population; Intervention recipient and content; Delivery personnel; Method of communication; Intensity and Complexity; Environment; Clinical outcomes) [4]. Accordingly, trials have been evaluated with different focuses, e.g. on changes in the organisation and delivery of care involving trained nurses [5], pharmacists [6], and call-centres providing home-based or telephone support or telemedicine [7,8], with mixed, but promising results. Ambulatory case management programmes "characterized by intensive post-discharge monitoring by a case manager (usually a nurse)" [4] have demonstrated positive effects on quality of life and mortality in three to six months follow-up, but the results are inconsistent for a longer follow-up [9-13].

Most studies have been conducted with acute patients enrolled in hospital or shortly after discharge rather than in primary care. Implementation of a primary care-based case management model in small-size office-based practices in Germany, which are led privately by primary care physicians, is problematical [14,15]. Unlike in many countries (e.g. UK and the US), practices in Germany do not have large teams or combinations of skill mix, i.e. they consist primarily of GPs (61% are solo practitioners [16]) and doctors' assistants (equivalent to a nursing role) [17]. German patients do not register with a single general practice and have free access to secondary care. Patients with complex chronic conditions like CHF receive regular follow-up in specialist care. Nevertheless, general practices are the primary caregivers and first point of contact for the majority of heart failure patients and represent a practical target for delivering case management interventions aimed at improved outcomes.

A comprehensive framework of chronic illness care suitable in primary care is the chronic care model (CCM), which aims to improve the care of patients by integrating a number of elements into a package designed to foster more productive interactions between trained, proactive teams and well-informed, motivated patients. The six elements that need to be addressed appropriately for providing high-quality care to patients within this model are delivery system design, self-management support, decision support, clinical information systems, community resources, and healthcare organisation [18].

The aim of this study was to evaluate such a case management model in comparison to usual care because no studies have applied a complex primary care-based model for heart failure patients in Germany.

## Methods

### Design

This study was part of an exploratory patient-randomised controlled trial (ISRCTN30822978) conceptualised as a phase II trial according to the framework of designing and evaluating complex interventions [14,15]. As such, qualitative methodologies were undertaken alongside the trial, to provide supplementary evidence [17,19]. The trial conformed to the principles outlined in the Declaration of Helsinki [20] and was approved by the institutional review boards of the local medical faculty of the university and the Medical Association of the federal state Baden-Württemberg in Germany. The full protocol of this trial has been published elsewhere [21]. The trial was an independent project within subproject 10 "Quality of Life" within the German "Competence Network Heart Failure", a nationwide research network that bundles the scientific expertise in a large-scale research network, sponsored by the Federal Ministry of Education and Research http://www.knhi.de[22]. Its aims include the coordination of basic and applied clinical research.

### Participants - Recruitment and Assignment

We invited general practitioners (GPs) to participate in the study through a single mail-out to primary care practices in one region of Northern Baden, Germany [21]. Interested GPs were eligible for participation if they were certified as primary care physicians or equivalent and practiced as statutory health insurance affiliated physicians. To be eligible for inclusion, doctors' assistants, all employed by the GP, had to have completed at least two years of their formal training (which consists of a three years practice-based, vocational training with part-time attendance at a vocational school) [21].

Eligible patients were adults with ascertained left ventricular systolic dysfunction (left ventricular ejection fraction of 45% or less), as described in detail elsewhere [21]. We obtained written informed consent from all participating health professionals and patients.

There is an assumed prevalence of patients with at least moderately impaired left ventricular function of 1% [1]. GPs were expected to include all eligible patients. Anticipating the difficulties with screening and patient inclusion, we estimated an average of 5 patients per GP (taking into account an average list size of 800 patients per GP in Germany) [23]. Case finding consisted of three strategies, i.e. brainstorming, opportunistic presentation for consultation by patients, and screening Electronic Medical Records using ICD-Codes (International Classification of Diseases) for (potential) heart failure (e.g. I 11* for Hypertensive Heart Disease, I 25* for CHD, I 40 for Myocarditis, I 42* for Cardiomyopathy, I 48* for Atrial Fibrillation or Flutter, I 50* for Heart Failure; * indicate further specifications).

Practices were recruited between June and November 2006 and practice staff enrolled patients between June 2006 and January 2007. Enrolment of patients with baseline assessment took place after obtaining informed consent during December 2006 and January 2007. Every participating GP sent a pseudonymised randomisation document for each patient to an external third party, the Coordination Centre Clinical Trials (CCCT), which supported the project in the conceptual and planning phase (e.g. creation of case report forms, statistical advice) and operational phase (allocation of patients, data management) of the trial. The statistician of the CCCT randomly assigned patients, based on a computer-generated list, to either the intervention or usual care on a weekly ongoing basis. Randomisation was stratified by a "Pocock algorithm" including the number and *status *of participating patients per practice and arm. *Status *was defined as whether patients participated in the previously conducted *train the trainer *study which ended in May 2006 [24]. After randomisation, each randomisation document was sent back to the practice with the result of the assignment. The nature of the intervention ensured that all participants were unblinded once assigned. The third party concealed intervention allocations from the practice-based intervention team until shortly before intervention commencement in February 2007. No interim analyses were conducted during the observation period, with the exception of data collection on heart failure medication for pharmacotherapy feedback. Neither the study statisticians nor the data monitoring committee saw personalised data or had any form of contact with study participants.

### Intervention

Patients randomised to the intervention received complex, structured case management by a trained doctor's assistant (DA) [21]: The design of the intervention addressed the 4 of the 6 elements of the CCM (delivery system design, self-management support, decision support, clinical information systems) [19]: DAs completed 6 hours of theoretical and practical training before conducting regular patient monitoring for 1 year by telephone (patients with NYHA functional status III or IV three-weekly versus I or II six-weekly) and by 3 home visits for all patients. DAs gave feedback of the results of the monitoring and screening to their employing GP. The programme included the use of a CPG, a patient leaflet according to the national CPG, booklets and tailored diaries. Further details of the intervention have been described previously [17,19,21]. Additionally, GPs received graphically depicted individual performance feedback on evidence-based pharmacotherapy (from data of baseline documentation) as described in a preceding trial [24].

For patients in the control (usual care) group, no case management was applied.

### Objectives

In a phase II trial [14,15], we explored whether a comprehensive case management intervention would improve patients' health-related quality of life (QoL), heart failure self-care, and patient-reported quality of care. Furthermore, we obtained data on hospital admissions and mortality.

### Outcome measures

For generic QoL we used the German version of the multidimensional SF-36 [25]. For disease-specific QoL we used the German version of the Kansas City Cardiomyopathy Questionnaire (KCCQ) [26], which has been shown to be a responsive instrument [27].

The SF-36 questionnaire consists of eight dimensions: Physical functioning, role functioning (physical), bodily pain, general health perceptions, vitality, social functioning, role functioning (emotional), and mental health. SF-36 scores are converted to a (T-) scale of 0 to 100, with *higher *scores indicating a higher QoL. The KCCQ quantifies several health status domains including physical limitations, symptoms (stability, frequency, and burden), self-efficacy, (mental) quality of life, and social function. Each scale is transformed to a score of between 0 and 100, with *higher *scores indicating superior health status. To summarise the multiple domains of health status quantified by the KCCQ, an overall summary score (KCCQ-os) has been developed that includes the physical limitation, symptoms, quality of life, and social function domains of the KCCQ: A mean five-point change in the scales of the SF-36 [28] and in the KCCQ-os [29] is regarded as clinically significant.

The European Heart Failure Self-care Behaviour Scale (EHFScBS) is a 12-item, self-administered questionnaire that covers items concerning the self-care behaviour of patients with heart failure, for example, daily weighing, fluid restriction, exercise or contacting a health care provider. Scores range from 1-5 (12-60), with *low *scores implying better self-care behaviour [30].

The self-administered extended Patient Assessment of Chronic Illness Care (PACIC-5A) instrument has been proven to be a practical, reliable and valid tool to measure quality of chronic care according to the elements of the CCM [31-34]. The PACIC-5A questionnaire asked patients 20 questions about important elements of chronic care, e.g. thinking about treatment choices, things that can be done to improve health, goals and treatment plans etc. The 5 subscales of the PACIC domain are patient activation, delivery system/practice design, goal setting/tailoring, problem solving/contextual, and follow-up/coordination. Six additional items measure given behavioural counselling according to the "5 A" principles (five subscales: assess, advise, agree, assist and arrange) regarding different chronic conditions according to the U.S. Preventive Taskforce recommendations [35,36]. All 26 items are scored on a 5-point Likert scale ranging from 1 (almost never) to 5 (almost always). Summary scores are generated for chronic care in accordance with the CCM and for the counselling according to the 5 A, with *higher *scores indicating a higher accordance.

### Data collection and management

GPs received an initiation visit by a study nurse including an introduction to the trial's investigator file. GPs collected and documented clinical data (history, current clinical status, lab results, ECG, detailed medication for assessment of guideline adherence etc.), discontinuation of the study by the patient, and death of the patient on pre-specified case report forms (CRFs) according to the Basic Clinical Dataset (BCD) of the Competence Network Heart Failure [22]. NT-proBNP levels at baseline were determined at the laboratory of the local university hospital using the Elecsys 2010 analyser from Roche Diagnostics, Germany. GPs also documented primary care activity by the number of practice attendances, referrals to a cardiologist, and hospital stays. The CRFs were sent to the responsible Coordination Centre Clinical Trials (CCCT), where data management was performed [22]. Parallel to the baseline and follow-up documentation after 12 months, patient-reported questionnaires were handed out by DAs. Patients were asked to return the questionnaires to the relevant DA in a pre-specified envelope within seven days. DAs then sent the questionnaires back to the study centre to enable the study nurse to monitor the progress of study documentation and intervene, e.g. by calling, if necessary. All questionnaires were then sent to the CCCT by the study team.

### Calculation of sample and effect size

The primary objective of this study was to explore the effectiveness of the CM intervention. Funding preconditions allowed a maximum total sample size of 200 eligible participants. Assuming an attrition rate of 30% due to loss to follow-up and death [1], we expected a total of 140 (2 × 70) patients. Given this sample size, an effect of about 0.45 could be detected using a one-sided t-test with a significance level of 5% and a power of 80%. Since this study was a pilot-trial, a one-sided t-test was chosen. The intervention would only be further investigated if it had a positive effect on QoL. For quality of life (on the physical functioning scale [28]) between the interventional and control arm at 12-months follow-up, an effect size of about 0.45 corresponds to a clinical difference [37] of about 9 points with a standard deviation of 20.6 suggested from available recommendations [28].

### Statistical methods

Descriptive methods were used for the analysis of the primary and secondary outcomes, including the calculation of appropriate summary measures of the empirical distribution (mean, standard deviation, median, minimum, maximum for continuous variables, and frequencies and percentages for categorical variables) as well as calculation of descriptive two-sided p-values for group comparisons. For continuous variables, analyses of covariance (ANCOVA) - adjusted for baseline score, age, gender, and practice type - were performed. Interaction terms for group and gender as well as for group and practice type were included in preliminary models. However, these effects were uniformly non-significant and were removed from subsequent models. For binary variables, logistic regression models were computed. Differences between the groups regarding variables of health care utilisation (e.g. practice attendances during follow-up) were analysed using t-test for normally distributed data and using Mann-Whitney-U-test otherwise. The statistical significance was set at 0.05. All analyses were performed using SPSS version 16.0.2 (SPSS Inc.).

### Ethical approval

This study was ethically approved by the Institutional review boards of the medical faculty of the University of Heidelberg and Medical Association of the state of Baden-Württemberg.

## Results

### Participants' flow and characteristics

**Figure 1:**
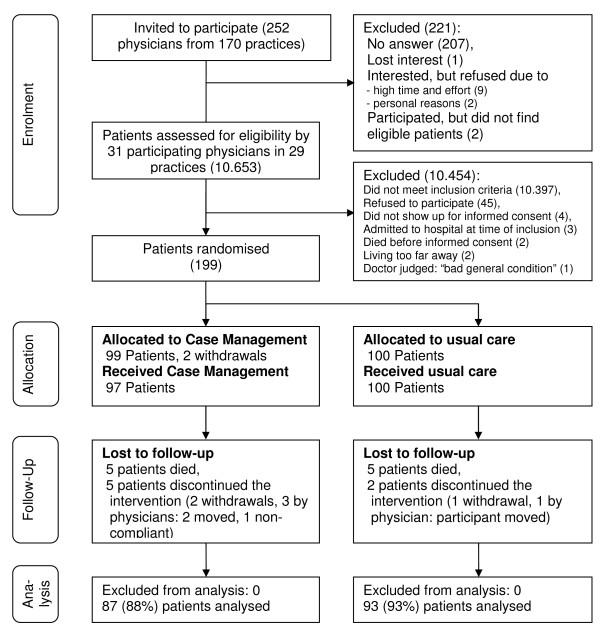
**Flow of participants through the trial**.

Figure 1 shows the flow of participating primary care physicians and patients through the trial. We approached a total of 252 physicians from 170 practices in a single mail-out in June 2006. Of these, 207 did not respond; 12 physicians expressed an interest but did not take part: 1 lost interest, 11 refused due to work load or personal reasons; and 2 physicians failed to find eligible patients. Therefore, 31 physicians from 29 practices participated. Between June and November 2006, these physicians screened 10653 patients for eligibility. Of these, 10397 failed to meet inclusion criteria, 45 eligible patients refused to participate, 4 did not show up for informed consent, 3 were admitted to hospital at time of inclusion, 2 died before informed consent, 2 lived "too far away" and 1 was judged by the treating physician as being unfit to participate.

The 31 participating physicians recruited 199 eligible patients: 99 patients were randomised to case management and 100 to usual care during December 2006 and January 2007. Once allocated to the case management, 2 participants immediately withdrew their consent rendering data collection at baseline impossible. Seventeen patients were ultimately lost to follow-up, 10 in the case management group and 7 in the usual care group, 5 patients died in each group (for details see Figure 1). Table 1 shows the characteristics of practices and staff. Table 2 shows the baseline characteristics for participants in the intervention and control group regarding socio-demographic, heart failure specific, comorbidity and treatment variables.

**Table 1:** Baseline characteristics of all 31 general practitioners (GPs) and 31 doctors' assistants (DAs) from 29 practices in Northern Baden.

Practice factors at baseline	(n = 29)
Practice type	
Single	10 (34)
Group practice	19 (66)

Location	
rural	14 (48)
suburban	5 (17)
urban	10 (35)

List size (patients per quarter*)	
0-1000	6 (19)
1001-1500	11 (36)
>1500	12 (45)

Medical staff	(n = 31)

No. of GPs per practice	
1	10 (32)
2	14 (45)
3	4 (13)
4	3 (10)

No. of DAs in practice	
1-3	11 (35)
4-6	16 (52)
7-11	4 (13)

Mean age of in years (SD)	
GPs	50.6 (9.0)
DAs	33.3 (9.6)

Female	
GPs	6 (19)
DAs	31(100)

Certification of GPs since mean years (SD)	16.3 (9.0)

DAs work experience since mean years (SD)	10.8 (9.1)

Participation of GP in	
Disease management programmes	29 (94)
Quality management programme	18 (58)

DA: Doctor's assistant; GP: General practitioner

Values represent number (percentages) of practices unless stated otherwise.

*In German ambulatory care, administration is organized by the Association of Statutory Health Insurance Physicians in 4 accounting periods per year, i.e. patients register in a practice (formally at a physician) in quarters

**Table 2:** Baseline comparison of intervention (n = 97) and control group patients (n = 100).

	Intervention group (n = 97)	Control group (n = 100)
Mean (SD; Range) number of patients per practice	3.2 (1.2; 1-6)	3.2 (1.7; 1-8)

Practice type		
single	35 (36.1)	38 (38.0)
group	62 (63.9)	62 (62.0)

List size (patients per quarter)		
0-1000	22 (22.7)	26 (26.0)
1001-1500	25 (36.1)	22 (22.0)
>1500	40 (41.2)	42 (42.0)

Male sex	69 (71.1)	74 (74.0)

Mean (SD) age (years)	70.4 (10.0)	68.9 (9.7)

Living alone	26 (26.3)	27 (27.0)

Social class*:	(n = 81)	(n = 84)
lower,	26 (32.1),	25 (29.8),
middle,	47 (58.0),	52 (61.9),
upper class	8 (9.9)	7 (8.3)

Participation of GP in Train the trainer-trial (GP received an)		

intensive educational intervention	14 (14.4)	18 (18.0)

control educational intervention	9 (9.3)	10 (10.0)

no educational intervention	74 (76.3)	72 (72.0)

NYHA-functional class (according to GP)		

I	1 (1.0)	5 (5.0)

II	63 (64.9)	67 (67.0)

III	33 (34.0)	27 (27.0)

IV	0	1 (1.0)

Mean (SD) LVEF	35.7 (7.5) (n = 83)	37.6 (6.7) (n = 88)

Main cause of CHF		

ischemic	46 (47.4)	47 (47.0)

non-ischemic	51 (52.6)	53 (53.0)

Mean (SD) duration (years) of CHF	6.2 (4.6) (n = 79)	6.8 (6.3) (n = 74)

Localisation of CHF		

Left	61 (62.9)	67 (67.0)

Left and right	33 (34.0)	31 (31.0)

Unknown	3 (3.1)	2 (2.0)

Cardiovascular interventions		

PTCA/Stent (any)	29 (29.9)	36 (36.0)

Bypass (any)	21 (21.6)	21 (21.0)

Pacemaker (right ventricular)	16 (16.5)	16 (16.0)

Pacemaker (biventricular)	7 (7.2)	8 (8.0)

ICD	11 (11.3)	21 (21.0)

Prosthetic heart valve (any)	5 (5.2)	7 (7.0)

Reanimation/Defibrillation	8 (8.2)	6 (6.0)

Medical conditions		

Atrial fibrillation	25 (25.8)	29 (29.0)

PAD	17 (17.5)	17 (17.0)

Cerebrovascular disease	22 (22.7)	16 (16.0)

Asthma	7 (7.2)	5 (5.0)

COPD	25 (25.8)	27 (27.0)

Depression	22 (22.7)	17 (17.0)

Cardiovascular risk factors		

Diabetes mellitus	32 (33.0)	35 (35.0)

Hypertension	78 (80.4)	78 (78.0)

Dyslipidemia	63 (64.9)	75 (75.0)

History of infarction before 60 years	18 (18.6)	16 (16.0)

Ex-/smoker (Ex: since at least 6 months)	38/9 (39.2/9.3)	42/14 (42.0/14.0)

Mean number (SD) of drinks per week	3.9 (5.5)	4.7 (9.2)

Creatinine-Clearance: Mean (SD) GFR (ml/min)**	71.6 (31.4) (n = 96)	71.6 (35.1)

Stage of renal dysfunction	(n = 96)	
GFR ≥ 60 ml/min**	54 (56.2)	57 (57.0)
GFR 30-59 ml/min**	39 (40.6)	37 (37.0)
GFR ≤ 29 ml/min**	3 (3.1)	6 (6.0)

Mean (SD) level of NT-pro-BNP [Median, IQR] in pg/ml	2222.5 (5282.2) [1010.5, 1750] (n = 94)	1828.6 (2914.9) [930, 1712] (n = 96)

Drugs at baseline included:		

ACE inhibitor *or *A2RA	91 (93.8)	95 (95.0)

β-blocker	70 (72.2)	84 (84.0)

ACE inhibitor/A2RA *and *β-blocker	66 (68.0)	80 (80.0)

Spironolactone/Eplerenone	32 (33.0)	26 (26.0)

Loop diuretics	60 (61.9)	59 (59.0)

Thiazide diuretics	34 (35.1)	41 (41.0)

Cardiac glycosides	25 (25.8)	26 (26.0)

Statins	57 (58.8)	58 (58.0)

Other lipid lowering drugs	11 (11.3)	14 (14.0)

Aspirin (100-300 mg/d)	38 (39.2)	43 (43.0)

Clopidogrel	5 (5.2)	5 (5.0)

Phenprocoumon (Vitamin K Antagonist: equivalent to Warfarin)	41 (42.3)	46 (46.0)

Values represent numbers (percentages) of patients unless stated otherwise.

*Social Class according to modified German Winkler-index [57] (lower class: 3-7; middle class: 8-14; upper class: 15-21)

**Estimation of the GFR according to the formula of Cockroft and Gault;

### Interventions

All of the participating DAs attended the training sessions, which were held at the local university.

Adjusted for loss to follow-up (5 patients died and 5 discontinued), 97 patients from the case management needed to receive 819 (100%) telephone monitoring sessions and 272 (100%) home visits according to their NYHA functional status: 786 (96.0%) and 269 (98.9%) of these case management interventions were finally conducted, respectively. Mean durations (SD; range) of the available overall 740 (90.3%) telephone monitoring sessions and the 3 available overall 256 (94.1%) home visits were 10 (5; 2-38), and 55 (14; 30-120), 53 (16, 18-90) and 51 (17; 21-90) minutes, respectively. The mean (SD; range) overall travel time for the home visits took 14 (15; 0-105) minutes. Reporting to the GP took 7 (6; 0-45), and 13 (6; 3-40), 15 (15; 2-75) and 12 (9; 3-50) minutes, respectively. Missed telephone monitoring sessions were scheduled in the last month of the follow-up period. Adding up the time for telephone monitoring, travel time, home visits, and reporting during the 12-month follow-up resulted in a mean (SD) overall time of 5.2 (2.0) hours per patient for patients in NYHA functional status I or II and 6.7 (2.4) hours per patient for patients in NYHA functional status III.

### Quality of life, behaviour change and quality of chronic illness care

The summary scores of the patient-reported outcomes are displayed in Table 3. Between group differences (95%CI) at the 12-month follow-up of the physical and mental component scale of SF-36 were minimal: -0.3 (-3.0; 2.5) and -0.1 (-3.4; 3.1). The *physical functioning *scale of the SF-36 (outcome for sample size calculation, see methods) showed a small, non-significant improvement in favour of the case management group (between group difference: 0.9 (95%CI -5.0; 6.8). With the exception of the *vitality *scale (6.6; 95%CI 1.8; 11.3; P = 0.008) there were no significant group differences in the SF-36 scales (data not shown in the table).

**Table 3:** Mean (SD) scores for groups for generic (SF-36) and disease-specific (KCCQ) quality of life, for self-care (EHFScBS), for chronic illness care (PACIC) and health counselling (PACIC-5A) at baseline and at 12 months' follow-up.

		Intervention (HICMan) group	No	Control group	No	Effect Mean Difference** (95% CI), Cohen's d	P value**
**SF-36**							

physical composite score	at baseline	36.4 (11.0)	78	36.9 (10.1)	82		
	
	at follow-up**	38.0 (8.6)	61	38.3 (8.6)	70	-0.3 [-3.0, 2.5], 0.04	0.857

mental composite score	at baseline	45.8 (11.9)	78	47.6 (12.8)	82		
	
	at follow-up**	46.5 (9.9)	61	46.6 (9.9)	70	-0.1 [-3.4, 3.1], 0.01	0.929

**KCCQ **overall summary score	at baseline	65.4 (22.6)	96	64.7 (22.7)	100		
	
	at follow-up**	68.0 (16.9)	87	66.3 (17.2)	93	1.7 [-3.0, 6.4], 0.10	0.477

EHFScBS*	at baseline	25.4 (8.4)	65	25.0 (7.1)	78		
	
	at follow-up**	21.2 (6.4)	65	24.8 (6.7)	78	**-3.6 [-5.7, -1.6], 0.55**	**0.001**

**PACIC **overall score	at baseline	3.2 (0.9)	89	3.2 (0.8)	97		
	
	at follow-up**	3.8 (0.7)	80	3.3 (0.7)	89	**0.5 [0.3, 0.7], 0.72**	**0.000**

**PACIC-5A **overall score	at baseline	3.2 (0.9)	89	3.2 (0.9)	97		
	
	at follow-up**	3.8 (0.7)	79	3.3 (0.7)	89	**0.5 [0.3, 0.8], 0.72**	**0.000**

*Scores range from 12-60; low scores imply better self-care behaviour.

**Based on analysis of covariance (ANCOVA) comparing results between groups at 12 months, adjusted for baseline score, age, gender, practice size (single vs. group-practice and list size) without interaction terms. All P values are descriptive.

KCCQ domains were compared for 87 intervention patients and 93 controls (93% of surviving intervention patients and 98% of surviving controls) (Figure 1 and Table 3). Between group differences (95%CI) for the KCCQ overall summary scores favoured CM: 1.7 (-3.0; 6.4). Significant (positive) time effects were found in the case management group for *self-efficacy *(6.5; 95%CI 1.6; 11.3) and *social limitation *(5.1; 95%CI 0.2; 10.1) (not shown in the table).

Heart failure self-care behaviour scores (EHFScBS) were analysable for 65 intervention patients and 78 controls (69% of surviving intervention patients and 82% of surviving controls), with significant group differences favouring the case management group (-3.6; 95%CI -5.7; -1.6, Cohen's d 0.55, P = 0.001, see Table 3).

Significant between group differences were found for quality of chronic illness care (PACIC) and behaviour counselling (PACIC-5A) (0.5; 95%CI 0.3-0.7; P = 0.000, and 0.5; 95%CI 0.3-0.8; P = 0.000), with moderate effect sizes (Cohen's d 0.7 for each summary score).

### Prescribing behaviour, hospital admissions and primary care activity

High prescription rates at baseline of drugs with good evidence for health outcomes (ACE inhibitor or A2RA, β-blocker, aldosterone antagonists) indicated an antecedent high guideline adherence by physicians; the increases in favour of the intervention group were non-significant (see Additional file 1).

For both groups, heart failure admissions during the pre-observation period were higher than during follow-up (36 vs. 35 cases): 18 heart failure admissions were observed in 11 patients in the intervention group while 9 were recorded by 7 patients in the control group, respectively (Additional file 1); this was a non-significant difference.

We analysed available primary care activity data during the observation period for 84 patients of the case management group and for 89 patients in the control group. The number of all-cause practice attendances was high at baseline and increased significantly during the follow-up in the intervention group (Mean (SD): 27.6 (16.1) vs. 23.9 (19.2), p = 0.02), but neither the number of practice attendances due to heart failure nor of contacts to cardiologists changed (Additional file 1).

## Discussion

### Summary of main findings

The intervention failed to improve the overall generic and disease-specific QoL. Nonetheless, we found significant improvements regarding patient-reported quality of care and CHF self-care. While showing low mortality rates (~5% in both groups) and decreasing heart failure hospital admissions, there was a significant increase in overall practice attendances in the intervention group but no change in contacts with cardiologists. The case management intervention was found to be feasible for the doctors' assistants to conduct. The following structured discussion also refers the exploratory character of the study and to the taxonomy and framework of chronic illness care as outlined in the introduction [4,18].

### Possible mechanisms of findings and their relation to other studies

The relatively high quality of life scores at baseline are similar to a large, multinational sample of outpatients with CHF (KCCQ-os: 66.2 ± 20.6) [38]. Our findings could partly reflect the antecedent high level of chronic care and self-care in our selected sample of physicians and patients with stable CHF (with ascertained left ventricular systolic dysfunction). This is supported by the finding of high evidence-based pharmacotherapy compared to landmark studies [39] and to recent clinical trials in secondary care [40], and by the level of chronic illness care and counselling [34], and good self-care (as shown in Table 3) [6,12,30]. However, given the social structure in Germany [41], the sample was skewed towards patients in lower socioeconomic classes but this is typically the case for primary care [42].

Observed mortality and heart failure hospitalisation rates were lower than reported previously [1,13], and a recent population study shows a trend of decreased mortality attributable to improvements in treatment and prevention [43]. Comparing our study with a meta-analysis regarding heart failure hospital admissions, rates for case management vs. usual care were 21.7% (18/83) vs. 10.4% (9/86) in our study (during 12 months follow-up) and 18.0% (94/523) vs. 29.4% (155/528) (with different follow-up periods from 3 to 12 months) [11]. While our study was not powered to this outcome, our findings resemble those of a recent larger trial, where an intensive disease management approach increased heart failure hospital admissions, where the authors concluded that the threshold may have been too low to admit patients to the hospital [13]. While GPs in our trial received an introductory clinical practice guideline, behaviour counselling and pharmacotherapy feedback, they were not specifically trained in clinical issues relating to thresholds for hospital admissions and we did not target physicians' behaviour regarding health care utilisation (referral or hospital admission). However, our intervention might have caused the unintended effect of increased hospitalisation (at higher costs). Whether our observation is an effect of the intervention cannot be concluded. The observed difference could have been by chance, due to the Hawthorne Effect among practice staff and in consequence undue admissions, or it could be a marker of higher quality of care. A recent review suggests that disease management interventions that do not involve efforts to improve physician care are less effective [44]. In our trial, the physician care targets were evidence-based pharmacotherapy (with little room for improvement) and counselling, and providing a basis for decision through CM with feedback of information to the physician.

Reviews of trials of case management programmes for patients with CHF have shown mixed results relating to effects on hospital admissions, mortality and QoL [9-11]. Furthermore, these studies have used a diversity of outcome instruments, which hinders comparability, e.g. disease-specific QoL has usually been measured with the Minnesota living with Heart Failure Questionnaire (MIlwHFQ), but generic QoL with the SF-36. Previous studies have shown variability in the complexity of case management (elements of the intervention, integration of care sectors, education and training of case managers, and patient empowerment): While overall positive effects on predominantly disease-specific QoL were found in the short term (3 to 6 months) follow-up [45-48], the results for longer follow-ups (9 months to 1 year) were predominantly neutral [5,12,49-52]: Typically, short-term positive effects on quality of life were observed in hospitalised and acutely ill patients, who started with low scores at baseline enabling the short-term effects in comparison to control [46-48]. However, our patient sample included stable chronic systolic heart failure and, in relation to their age and disease, relatively high quality of life scores at baseline [28,52]. Regarding generic QoL, our results suggest that an effect size of 5 points would not be reached irrespective of the power of the sample size. Regarding disease-specific QoL, as the upper CI exceeds 5 points (KCCQ-os, see Table 3), results can be regarded as inconclusive. Nevertheless, QoL did not decrease in both groups after 1 year, which may be seen as a treatment (and observational) effect, as the natural course of patients with CHF and normal population shows a decline in QoL [53].

Two previous randomised controlled trials with the same (1 year) follow-up period and similar sample sizes, sex and age profiles showed clinically relevant effects on QoL and hospital admissions [4,5,12]. They differed from our study in that the interventions were conducted mainly in a secondary care setting and the *targeted *subgroups of heart failure patients included patients in more acute phases, with a higher functional and objective disease severity (exclusively NYHA III and IV patients, stronger impaired systolic function). While comorbidity was similar, our sample showed higher rates of diagnostic measures and causal therapies indicated by cardiovascular interventions (PTCA/Stent: 33% vs. 15%, CABG: 21% vs. 24%, Pacemaker: 24% vs. 9%) (Table 2, [12]). However, the main novel aspect of our trial relates to the *staff who delivered *case management, the *intervention content*, the *methods of communication*, and the *intensity and complexity of the intervention *[4]: Doctors' assistants and GPs vs. cardiologists and specialised nurses were involved. While all programmes had a similar frequency of face-to-face contacts, the quality and the intensity of the medical care (e.g. investigations [12]) and educational content were higher in other studies [5,12,54]. Knowing these contextual differences with regard to training level and skills of staff, our intervention included a provider intervention (training of doctors' assistants and introduction for GPs) and used an elaborated standardised case management concept enabling doctors' assistants to deliver case management. It addressed 4 of the 6 domains of the chronic care model [18]: delivery system design (giving a doctor's assistant a new role), self-management support (by counselling and the questions included in the monitoring lists), decision support (by patient and physician guidelines and pharmacotherapy feedback) and clinical information systems (a registry, patient booklets).

Heterogeneous results regarding QoL have been found in other primary care-based patient populations: In cardiovascular high risk patients, improvements in many scales of generic QoL have been found in primary care-based interventions [52,55]. However, either the baseline level was low leaving more room for improvement (physical functioning) [52], or, if baseline was high, the relative improvements (in a big sample) were small, and probably not clinically relevant [55].

### Strengths and limitations of the study

The strengths of the study are that many aspects of the trial promoted high internal validity. Randomisation was concealed and conducted by a third party; there were reasonably equivalent groups of patients at baseline (with the exception of differences in the prescription rates of β-blockers) and good follow-up of patients (93-95% for the potential primary outcome). However, a weakness is that it was impossible to blind providers to treatment group, which may have biased their activity as well as patient responses to questionnaires. Furthermore, randomisation was performed at the patient level and all GPs received an introduction to guideline-oriented management of CHF and counselling, which may have led to some contamination between the interventions. While a cluster randomised controlled trial might have mitigated contamination, we believe any bias was limited because the main aspects of the intervention were performed by doctor's assistants and instruments (monitoring lists, screening instruments, booklets, patient information) and were delivered to each individual patient. If there was a bias, we believe that the consequence is that the measured effect of the intervention may be underestimated.

The baseline characteristics of GPs and the pharmacotherapy profile indicate a non-representative sample of physicians. Moreover, group practices dominate our sample while, in Germany, solo practices are most common (64.1%) [56]. Also, participating GPs showed some signs of above average performance and early adoption of new methods of practice, for example participating in disease management programmes. Furthermore, some of the GPs and their patients participated in a preceding trial aimed at optimisation of heart failure care in general practice [24]. However, baseline prescription rates were high also in that study, so it is probably the selection of patients with ascertained left ventricular systolic dysfunction and subsequent prescription of evidence-based pharmacotherapy that is observed.

Considering these aspects of internal and external validity, further evaluation in a larger representative sample for robust data is needed to draw definitive conclusions on the effectiveness on QoL, mortality and health service utilisation.

## Conclusions

This phase II trial demonstrates some positive effects of primary care-based case management on self-care and quality of care in chronic, stable patients with CHF. The approach was seen as feasible by practice staff [17,19].

Improved quality of chronic care and CHF self-care could be regarded as important and targeted outcomes for an intervention in elderly, multimorbid patients with CHF in primary care. The CM intervention was successfully adapted for primary care settings for this patient group and while the approach could be augmented by the use of exercise training programmes which are generally known to be effective, these are not always feasible. It is possible that patients with more advanced CHF might profit from a CM model approach, with pre-defined involvement of specialist and integrated care approaches, including (post-) discharge approaches.

## Abbreviations

A2RA: Angiotensin-2 receptor antagonist; ACE: Angiotensin converting enzyme; CCCT: Coordination Centre Clinical Trials; CCM: Chronic Care Model; CHD: Coronary heart disease; CHF: Chronic (systolic) heart failure; CI: Confidence interval; COPD: Chronic obstructive pulmonary disease; CPG: Clinical practice guideline; EHFScBS: European Heart Failure Self-care Behaviour Scale; GFR: Glomerular filtration rate; GP: General practitioner; HICMan: Heidelberg Integrated Case management; ICD: Implantable cardioverter defibrillator; IQR: Inter Quartile Range; KCCQ: Kansas City Cardiomyopathy Questionnaire; LVEF: Left ventricular ejection fraction; LVSD: Left ventricular systolic dysfunction; NT-proBNP: N-terminal Brain Natriuretic Peptide; NYHA: New York Heart Association; OR: Odds ratio; PACIC: Patient Assessment of Chronic Illness Care; PAD: Peripheral arterial disease; PC: Primary Care; PHQ-9: Depression module of the Patient Health Questionnaire; PTCA: Percutaneous Transluminal Coronary Angioplasty; QoL: (Health-related) Quality of life; RCT: Randomised controlled trial; SF-36: MOS 36-item short-form health survey.

## Competing interests

The authors declare that they have no competing interests.

## Authors' contributions

FPK, TMT and JS designed the study. CUK, KH and FPK analysed the results. All authors interpreted the results. FPK wrote the manuscript, and is the guarantor. All authors contributed to writing revisions and approved the final manuscript.

## Supplementary Material

Additional file 1 **Guideline Adherence, mortality, hospital admissions, practice attendances and referrals to cardiologist at baseline or during 12 months' pre-observation period and at or during 12 months' follow-up.** Frequencies (percentages) are shown for groups unless stated otherwise. Click here for file
