# Supplementary material for: Case management for patients with chronic systolic heart failure in primary care: The HICMan exploratory randomised controlled trial
Source: Trials. 2010 May 17;11:56. doi: 10.1186/1745-6215-11-56 (PMC2882359; doi:10.1186/1745-6215-11-56)
Supplement: Additional file 1 — Guideline Adherence, mortality, hospital admissions, practice attendances and referrals to cardiologist at baseline or during 12 months' pre-observation period and at or during 12 months' follow-up. Frequencies (percentages) are shown for groups unless stated otherwise. [file 1745-6215-11-56-S1.DOC]

**Table 4.** Guideline Adherence, mortality, hospital admissions, practice attendances and referrals to cardiologist at baseline or during 12 months’ pre-observation period and at or during 12 months’ follow-up.

|  | Baseline or pre-observation period* | | | | Follow-up | | | | Effect |
| --- | --- | --- | --- | --- | --- | --- | --- | --- | --- |
|  | Intervention (HICMan) group | No | Control group | No | Intervention (HICman) group | No | Control group | No | Odds ratio (95% CI); P value |
| Guideline Adherence |  | 97 |  | 100 |  | 87 |  | 93 | ***/ **** |
| ACE inhibitor *or* A2RA | 91 (93.8) |  | 95 (95) |  | 80 (92) |  | 81 (88) |  | 3.8 (0.8, 18.5); 0.1 |
| β-blocker | 70 (72.2) |  | 84 (84.0) |  | 67 (77) |  | 75 (81) |  | 1.7 (0.6, 5.3); 0.3 |
| ACE inhibitor/A2RA *and* β-blocker | 66 (68.0) |  | 80 (80) |  | 63 (72) |  | 67 (73) |  | 2.5 (0.9, 7.2); 0.09 |
| Spironolactone/Eplerenone | 32 (33.0) |  | 26 (26) |  | 34 (39) |  | 30 (32) |  | 0.9 (0.3, 2.7); 0.9 |
|  | | | | | | | | | |
| Death of any cause (cardiac / non-cardiac / unknown cause) |  |  |  |  | 5 (2/2/1) | 97 | 5 (3/1/1) | 100 | 1.1 (0.3, 3.8); 0.92*** |
|  | | | | | | | | | |
| No of patients with ≥ one heart failure hospital admission | 23 | 87 | 29 | 93 | 11 | 97 | 7 | 86 | 1.7 (0.6, 4.7); 0.29 |
| Hospital admissions |  |  |  |  |  |  |  |  | GLM; p-value |
| any cause | 56 | 97 | 74 | 100 | 40 | 87 | 34 | 91 | 0.44 |
| due to heart failure | 36 | 87 | 35 | 93 | 18 | 83 | 9 | 86 | 0.16 |
|  | | | | | | | | | |
| Mean (SD) and median [MD] practice no. of attendances |  |  |  |  |  |  |  |  | Mann-Whitney-U-test; p-value |
| any cause | 26.9 (17.6) | 94 | 23.1 (15.2) | 98 | 27.6 (16.1) [26] | 84 | 23.9 (19.2) [18] | 90 | **0.02** |
| due to heart failure | 14.8 (14.5) | 94 | 12.4 (11.8) | 98 | 14.9 (13.9) [10] | 97 | 12.4 (15.0) [9] | 89 | 0.93 |
| Mean (SD) and median [MD] no. of contacts to cardiologist during pre-observation period | 2.2 (2.1) | 97 | 2.1 (2.1) | 100 | 1.7 (1.7) [1] | 87 | 1.8 (2.1) [1] | 93 | 0.75 |

*12 months period before baseline

**Based on analysis of covariance (ANCOVA) comparing results between groups at 12 months

*** Based on logistic regression model comparing number of outcomes between intervention vs. control group

****Adjusted for baseline score, age and gender; adjusted for New York Heart Association functional class, and β-blocker at baseline.

All P values are descriptive. Frequencies (percentages) are shown for groups unless stated otherwise.
